# Potential role of a reduced nephron endowment and impaired kidney functional reserve in the pathogenesis of hypertensive disorders of pregnancy

**DOI:** 10.1007/s40620-025-02330-5

**Published:** 2025-07-24

**Authors:** Silvia Visentin, Pierpaolo Zorzato, Erich Cosmi, Maria Cristina Mancuso, Letizia Dato, Francesco Cavallin, Gianluigi Ardissino

**Affiliations:** 1 Department of Women’s and Children’s Health, University of Padua, Padua, Italy; 2 Center for HUS Control, Prevention and Management, Fondazione IRCCS Ca’ Granda Ospedale Maggiore Policlinico, Via Francesco Sforza 35, 20122 Milan, Italy; 3 Independent Statistician, Solagna, Italy

Hypertensive disorders of pregnancy (HDP) are important causes of severe morbidity, long-term disability and death among both pregnant women and their foetuses. Maternal complications include preeclampsia, eclampsia, placental abruption, kidney failure, liver failure, cerebrovascular accidents, and haemolysis, elevated liver enzymes, low platelet count (HELLP) syndrome. Stillbirth, intrauterine growth restriction, low birth weight and cardiovascular disease later in life may affect the newborn’s outcome.

African descent, a history of previous hypertensive disorder of pregnancy, metabolic, renal or autoimmune maternal diseases, nulliparity, a positive family history for HDP, multifoetal gestation and a maternal age > 35 years are well-known risk factors for HDP. Except for pregestational hypertension, the remaining conditions, including gestational hypertension and preeclampsia, have unclear pathophysiology. Prevention, early diagnosis and timely and appropriate treatment are the mainstays of management [S1].

During pregnancy, plasma volume expansion occurs through the stimulation of renal sodium and water retention. These physiological changes increase renal blood flow and decrease oncotic pressure, thus contributing to an increase in the glomerular filtration rate (GFR). In humans, this adaptation is reported to occur as early as the first weeks after conception and is usually sustained until the end of gestation, but available data show considerable individual variation [1]. As a consequence, in physiological, uncomplicated pregnancies, a decrease in serum creatinine (sCr) is observed as early as the 4th gestational week [1].

Considerable evidence points to a key role of kidneys, particularly nephron endowment, in the pathogenesis of HDP. First, patients with a solitary kidney have an increased risk of HDP, as do patients with a reduced nephron mass due to preterm birth, low birth weight, or any type of kidney disease [2, 3]. Most patients with HDP are normotensive before and after pregnancy but remain at increased risk of early development of chronic hypertension and chronic kidney disease (CKD) later in life, similarly to subjects with low nephron endowment [4, 5]. Moreover, low nephron endowment is a familial trait, as are HDP.

Unfortunately, measuring the nephron number is technically unfeasible except postmortem; thus, the hypothesis that HDP are mainly due to reduced nephron endowment must rely on indirect evidence. Serum creatinine is not a good marker of nephron number, as it typically rises when more than 50% of the functioning kidney tissue is damaged. Moreover, it should be emphasized that sCr is not even currently included in the routine monitoring of physiological pregnancies.

In a pilot study herein reported, undertaken to guide a future prospective analysis, we investigated kidney function in 66 previously apparently healthy women with singleton pregnancies (Table 1), all of whom had sCr levels within the normal range during the first trimester and no proteinuria. The identification of patients with HDP followed the ISSHP 2021 recommendations [6]. The control group consisted of women referred to our hospital with uncomplicated pregnancies, matched to the cases for maternal and foetal characteristics. We found that women (*n* = 33) who subsequently developed HDP, at a median gestational age of 35 weeks (IQR 25–40), had a median sCr level of 57 µmol/L (IQR 51–58) compared with 43 µmol/L (IQR 38–48) in the control group (*n* = 33) (*p* < 0.0001) (Fig. 1A). Although statistically significant, the age difference between the two groups is modest (Table 1) and probably cannot explain the difference observed in sCr levels between cases and controls. Adjusting for age by a linear regression model, the difference in sCr levels remained statistically significant (mean difference 11 µmol/L, 95% CI 7–15; *p* < 0.0001). Receiver operating characteristic curve analysis for the prediction of HDP based on sCr levels in the first trimester revealed an area under the curve of 0.86 (95% CI 0.76–0.96) and suggested a sCr threshold of 49.5 µmol/L, with a sensitivity of 0.79 (95% CI 0.61–0.91) and a specificity of 0.88 (95% CI 0.72–0.97) (Fig. 1B). The variation of sCr levels during pregnancy was further investigated using a linear mixed model, with time and group (cases vs. controls) as fixed terms, and the patient as random term. The model suggested an increasing trend in sCr levels during pregnancy in cases who later developed preeclampsia (*p* < 0.001; Supplementary Fig. 1).

**Table 1:** General characteristics of cases and controls in the first trimester of pregnancy

	Cases (*n* = 33)	Controls (*n* = 33)	*p*-value
Age (years)^a^	32 (29–39)	29 (26–35)	0.04
Previous pregnancy^b^	11 (33%)	14 (42%)	0.61
BMI (kg/m^2^)^a^	23.9 (21.0–26.8)	24.0 (23.0–27.0)	0.60
Proteinuria (g/L)^a^	0.15 (0.14–0.16)	0.17 (0.10–0.21)	0.71
Albuminuria (mg/g)^a^	8.7 (8.0–9.1)	7.9 (6.6–9.2)	0.43

^a^Median (interquartile range)

^b^Number (%)

*BMI* body mass index

**Fig. 1:**
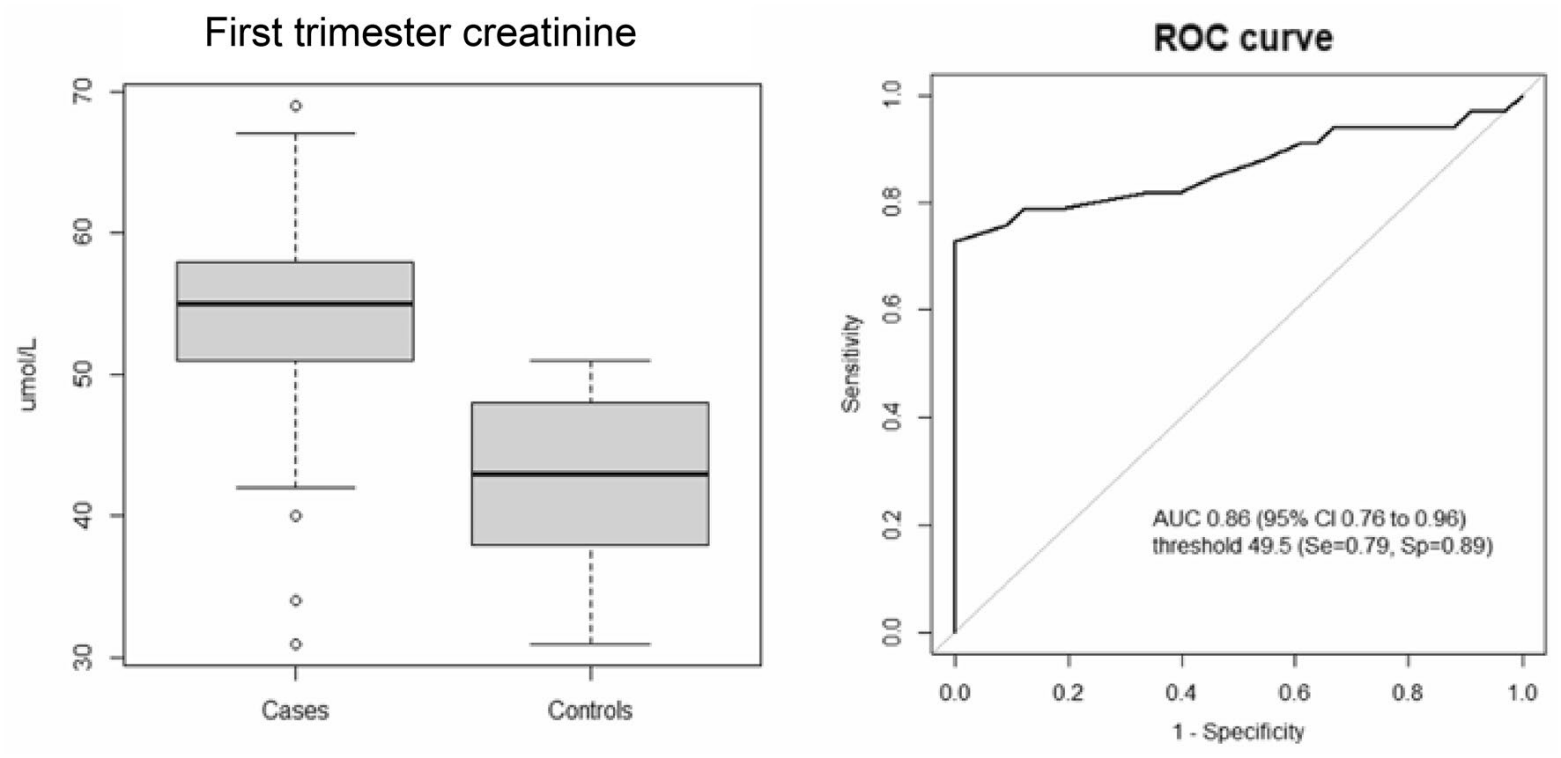
Serum creatinine levels in the first trimester of pregnancy in cases and controls (box and whiskers plot, **A**) and receiver operating characteristic (ROC) curve for the prediction of hypertensive disorders of pregnancy (HDPs) based on creatinine levels (umol/L) in the first trimester (**B**). *AUC* Area under the curve, *CI* Confidence interval, *Se* Sensitivity, *Sp* Specificity

Pre-conception sCr levels were not available in this retrospective cohort, but a prospective study is being planned in which pre-conception kidney function will be determined, together with cardiovascular assessment.

It is usually held that healthy subjects have an average of 1,000,000 nephrons per kidney. However, more detailed investigations have revealed that as much as 7% of the general population has fewer than 500,000 nephrons, and that 3.5% has fewer than 300,000. A low nephron endowment, which is genetically determined, may have several consequences for an individual’s health, such as an increased risk of hypertension, nephro-angiosclerosis, increased risk of drug-induced nephrotoxicity and kidney failure [3].

Serum creatinine alone cannot reveal this low nephron endowment unless a stressful event occurs: individuals with low nephron mass eventually exhibit an increase in sCr during acute dehydration, infection or increased functional demand [5]. As mentioned above, healthy pregnancy is associated with an increase in kidney functional demand, corresponding to an increase in the GFR as high as 40% [1].

We speculate that the lack of decrease in sCr among women who later developed HDP may be interpreted as impaired kidney functional reserve due to low nephron endowment. This hypothesis is supported by several observations leading to a central pathophysiological role of the kidney in HDP [7, 8]. First, blood pressure is mainly regulated by the kidney, and most kidney diseases are associated with hypertension. In addition, proteinuria, a characteristic of preeclampsia, clearly indicates kidney involvement. Finally, the neurological picture observed in eclampsia patients can be ascribed to posterior reversible encephalopathy syndrome, which, in turn, is mainly observed as a complication of kidney diseases. Moreover, women who develop HDP are at increased risk for developing hypertension and CKD later in life [4].

Our preliminary findings further support that women who develop HDP have low nephron endowment [8, 9].

As mentioned above, measuring the nephron number is technically unfeasible, but the lack of a decrease in sCr in the first trimester of pregnancy may provide indirect evidence of this condition.

We are currently planning a study that aims to address this gap by evaluating kidney function from the pre-conception period through the puerperium. In addition to sCr, other markers of kidney damage and endothelial dysfunction will be included. We also plan to include a comprehensive assessment of the maternal cardiovascular system to provide further insight into other maternal cardiac and renal factors contributing to the development of HDP.

## Supplementary Information

Below is the link to the electronic supplementary material.

Supplementary file1 (TIFF 531 KB)

Supplementary file2 (DOCX 14 KB)

## Data Availability

Data are available on reasonable request.
