# Supplementary figures and images for: Potential role of a reduced nephron endowment and impaired kidney functional reserve in the pathogenesis of hypertensive disorders of pregnancy
Source: J Nephrol. 2025 Jul 24;38(8):2029–31. doi: 10.1007/s40620-025-02330-5 (PMC12630276; doi:10.1007/s40620-025-02330-5)

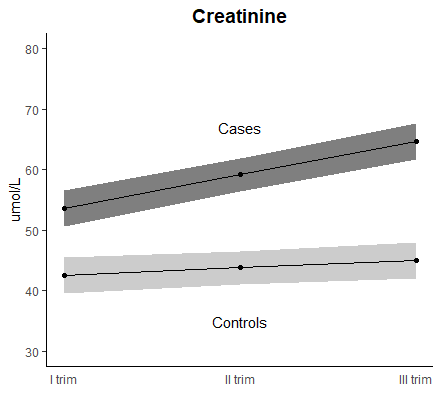

Supplement: Supplementary file 1 — Supplementary file1 (TIFF 531 KB) [file 40620_2025_2330_MOESM1_ESM.tiff]
