# Supplementary material for: Potential role of a reduced nephron endowment and impaired kidney functional reserve in the pathogenesis of hypertensive disorders of pregnancy
Source: J Nephrol. 2025 Jul 24;38(8):2029–31. doi: 10.1007/s40620-025-02330-5 (PMC12630276; doi:10.1007/s40620-025-02330-5)
Supplement: Supplementary file 2 — Supplementary file2 (DOCX 14 KB) [file 40620_2025_2330_MOESM2_ESM.docx]

**Supplementary Material**

Supplementary Figure 1. Estimated trend of serum creatinine levels during pregnancy in cases and controls. Shaded areas represent the 95% confidence intervals.

**Supplementary references:**

S1. WHO Recommendations for Prevention and Treatment of Pre-Eclampsia and Eclampsia. Geneva: World Health Organization; 2011. PMID: 23741776.
